# Efficacy and efficiency of fracture liaison services to reduce the risk of recurrent osteoporotic fractures

**DOI:** 10.1007/s40520-021-01844-9

**Published:** 2021-05-28

**Authors:** M. K. Javaid

**Affiliations:** Nuffield Department of Orthopaedics, Rheumatology and Musculoskeletal Sciences, University of Oxford, Oxford, UK

**Keywords:** Fracture liaison service, Osteoporosis, Quality improvement

## Abstract

**Background:**

Acting to prevent the next fracture after a sentinel fracture is support by the evidence base and brings benefits for patients, clinicians and healthcare systems. However, more patients after a fragility fracture remain untreated and vulnerable to future potentially life-changing fractures. Fracture liaison services (FLS) are models of care that can close this care gap.

**Methods:**

A narrative review of the key evidence for the efficacy and effectiveness of FLS was performed

**Results:**

There are few randomised control trials of FLSs and none with fracture as the primary outcome. Several observational studies have also demonstrated reductions in fracture, but most were limited by potential bias. Several studies have highlighted that not every FLS is automatically effective.

**Conclusion:**

Further research should focus on implementing effective FLS using published standards and only then exploring impacts on patient outcomes such as refracture rates.

## Introduction

**Fig. 1:**
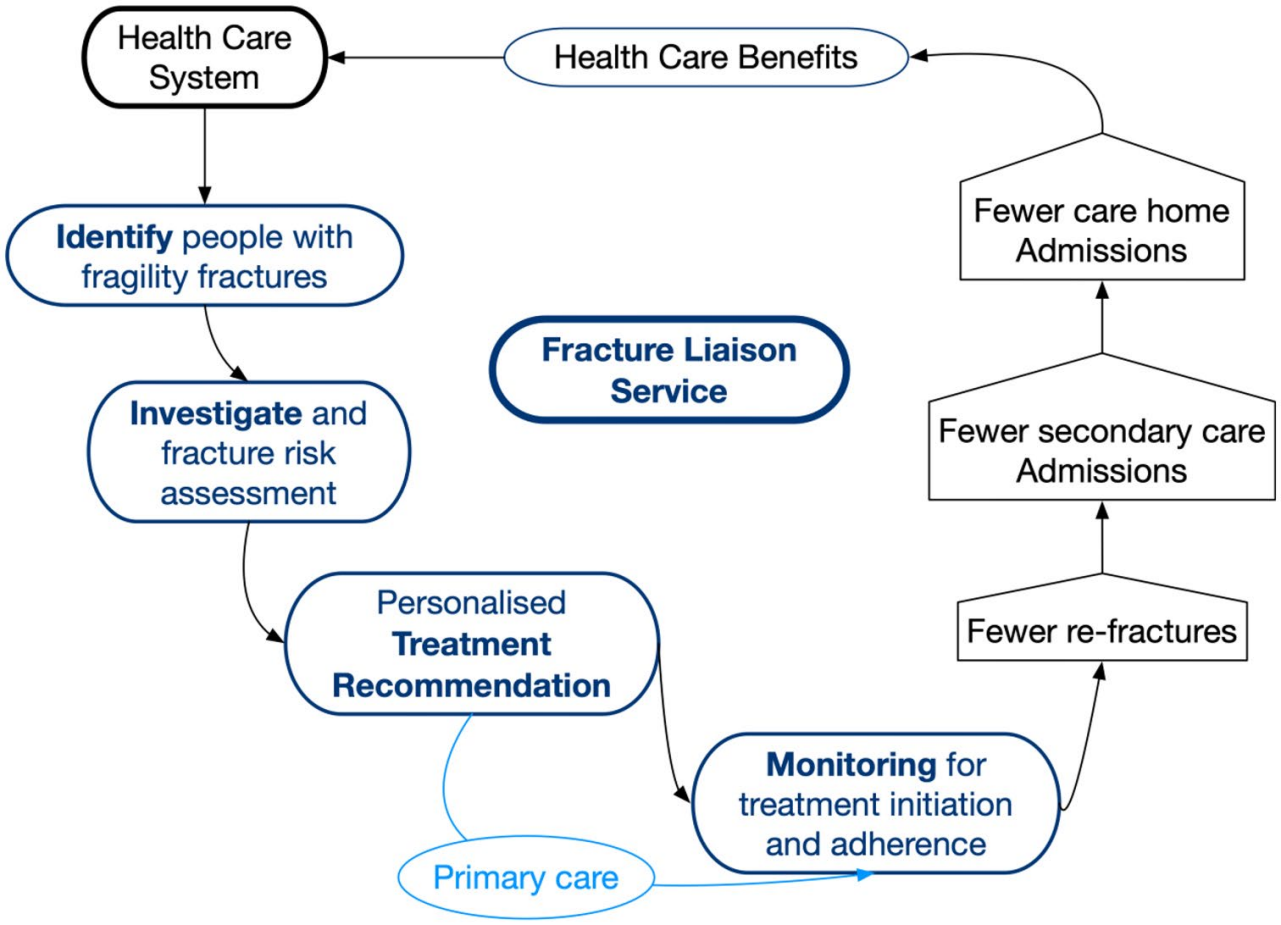
Key components of an FLS

A fragility fracture is a significant event for patients and their family, leading to a substantial loss of quality of life and an increase in mortality [1]. Fragility fractures are a recognised significant risk factor for further fractures [2, 3]. Healthcare systems are now beginning to recognise the benefits of secondary fracture prevention [4] and prioritise secondary fracture prevention above primary prevention and fall prevention, where the return on investment of healthcare resources may be less. Despite effective treatments to reduce fracture risk, less than 50% of patients receive effective secondary fracture prevention after a fragility fracture [5, 6]. To address this care gap, several initiatives have been published to improve clinical services by implementing fracture liaison services (FLSs) [7–14]. Initiated in the 1990s by Drs McLellan [15] and Gallacher [16], an FLS works is a team of healthcare professionals that systematically identifies, investigates, recommends treatment and then monitor patients to optimise adherence to evidence-based interventions to reduce fracture risk (Fig. 1) [17]. The International Osteoporosis Foundation was the first international organization that capitalized on FLS through its very comprehensive Capture the Fracture programme, launched in 2012. Several hybrid service solutions such as Osteoporosis Liaison Services (OLS), orthogeriatric services (OGS) have since evolved and fit into the broad category of Post Fracture Care (PFC) services, of which FLS is an example.

## Efficacy of FLS

Large scale randomised trials have demonstrated the clinical effectiveness of anti-osteoporosis medication to reduce fracture risk significantly [18–25]. Further, analyses of routine health data have demonstrated the potential for anti-osteoporosis medications to reduce fracture risk in the real-world setting [26]. The scale of fracture reduction depends on the fracture site with the most significant reductions seen for vertebral fractures, then hip and non-spine/nonvertebral fracture sites [27]. Translating these benefits to an FLS setting depends on many factors. The first step is an identification strategy enriched for patients at moderate or high risk. This may include using age thresholds, such as over 50, 65- or 75-year-olds or sites of fracture, such as the hip, spine, femur, humerus, pelvis and ribs. The objective is to find enough moderate/high-risk patient and fewer low-risk patient who after assessment, do not qualify for treatment. The relevance of the level of trauma for case-finding has been challenged by the evidence that patients with fractures from high trauma are at greater risk of future osteoporotic fractures [28], BMD is low in patients who have fractures after major trauma injuries [29] and anti-osteoporosis medications reduce fracture after simple falls as well as after high-trauma injuries [30]. Most guidelines would exclude fracture of the scaphoid, face, skull and digits. Inclusion of ankle and metatarsal/metacarpal fractures could be considered after evidence that identification of higher risk fracture sites has been optimised.

The second step is ensuring that patients identified by the FLS are assessed rapidly so eligible patients are recommended specific anti-osteoporosis therapy that reflects their baseline risk of fracture. After a sentinel fracture, the risk of refracture is high [31], and up to 50% of the 10 year risk is compressed into the first two years post-fracture [32], the imminent fracture risk [33]. Different anti-osteoporosis treatments have differing relative potencies [34, 35] as well as time to onset [36]. This evidence needs to be in balance with national reimbursement when personalising treatment recommendations. The third step is ensuring eligible patients who are recommended AOM, start them promptly, given the potential delay between treatment onset and benefit [36], and adhere.

## Effectiveness of FLSs

To date, no randomised control trials have demonstrated the superiority of FLS in reducing fracture risk, the primary outcome of a service.

Clinical trials with surrogate outcomes such as DXA testing and treatment initiation and adherence in this area were led by the late S.Majumdar. His team demonstrated increases in diagnosis and post-fracture treatment in series of randomised controlled trials with blinded ascertainment of outcome in adults with upper limb fractures in the emergency room setting, [37, 38]. Disappointingly, longer term follow-up demonstrated an unexpected lower adherence to therapy at 24 months, highlighting the need for adequate follow-up for FLSs [39]. Limitation of these trials include exlusion of patients with cognitive impairment or patients requiring inpatient care, limiting generalisability.

Randomised trials may be considered inappropriate given the lack of equipoise that FLS are effective from policy, clinical and patient perspectives. Further, FLS trials would likely need to be cluster randomised to limit contamination within sites and for recruited sites to agree not to initiate an FLS during the trial duration, even within a step wedge design. The estimated sample size to demonstrate a 25% reduction in refractures at 2 years with an expected refracture rate of 12% would exceed 15,000 participants using a cluster design and take over 4 years to deliver, challenging the value of information of such research. Finally, an FLS is a complex intervention requiring multiple levels of engagement within the existing healthcare systems and has to adapt to changes in local and national changes in health delivery as well as the availability of new AOMs. Ensuring intervention fidelity would be difficult.

Given the challenges for running clinical trials, observational studies have been used to examine the effectiveness of FLSs. There are three broad types of study. The most typical method is to compare differences in fracture rates before and after the introduction of a fracture prevention service. One of the first studies was from the South California S Permanente, USA. The introduction of the Kaiser model led to a 37% reduction in hip fracture rates and an associated × 2–6 increase in DXA rates and prescribing rates [40]. However, there was no contemporaneous control arm and only three pre-intervention data points to model the expected exponential fracture rate trajectory accurately. In terms of the intervention, the Kaiser secondary fracture prevention model required an integrated information system linking hospital admissions, primary care physician visits, bone density scanning and pharmacy dispensing to case find, assess, initiate and detect discontinuation and included primary prevention, which limits generalisability. To overcome the issue of contemporary controls, an interrupted time series of analysis compared outcomes across 11 healthcare centres in the UK that introduced orthogeriatric and FLS services at different time points to try to overcome the issue of secular trends [41]. While a significant reduction in mortality was demonstrated, no effect on refracture rates was seen with almost 34,000 sentinel hip fractures. A significant limitation of this study was the FLS interventions typically did not include a monitoring component [42]. The Ontario Fracture Clinic screening program also used an interrupted time series methodology to compare BMD testing, treatment initiation and adherence 1 year after fracture across in hospitals with a PFC program and non-intervention hospitals [43]. From 147,071 individuals with an index fracture, BMD testing improved from 17% pre-intervention to 20.9% post-intervention with no change for individuals who were cared for by the non-intervention hospitals (14.9%). However, while there was an increase in treatment initiation, the proportion with adequate persistence fell from pre- to post-intervention from 45.8% to 40.% at PFC sites. The reduction in adherence was greater in non-intervention sites. The Swedish four hospitals study used electronic health records to compare the incidence of hip, clinical spine, humerus, radius and pelvis fractures before and after the implementation of different types of FLS [44]. Two hospitals instituted FLSs that included referring identified patients for FRAX and DXA and routine inpatient or community zoledronate or denosumab post-hip fractures. Following 21,083 sentinel fractures, there was an 18% reduction in recurrent major osteoporotic fractures after the intervention period in FLS hospitals with no change in fractures rate observed in the non-FLS hospitals. The largest benefit was seen in those aged 82 years and over.

Another study methodology is to use osteoporosis drug therapy rates to predict changes in fracture rates. The Glasgow Fracture Liaison Service, UK, used eight-year audit data to inform a Markov model of secondary fracture prevention using published trials [45]. The model predicted a 7% reduction in fractures at 5 years. However, all patients were assumed to remain on treatment for 5  years despite no active monitoring programme and a stable 5 year off-treatment was assumed. The Toronto study published cost-effectiveness results on treatment initiation and 1 year adherence rates in coordinator vs non-coordinator settings and demonstrated significant cost savings [46]. Again, this study did not directly measure refracture rates, estimated treatment initiation and adherence in the non-coordinator setting and used fracture reduction rates based on clinical trials.

The final method is to use observed fracture rates from patients who did or did not attend a specialist service. The CONCORD study, Australia, demonstrated an 80% reduction in clinical fractures over 5 years [47]. This difference in clinical fracture rates is unexpected and far exceeds findings from randomised control trials. Using patients who did not attend the specialist clinic as the ‘comparator’ group would result in a significant immortal time bias. An immortal time bias is where patients are not able to able to experience the outcome during a portion of follow-up as part of the study design. A more general selection bias occurs, as those who attend a PFC service are more likely to be healthier and have fewer co-morbidities. It is also noted that only 20% of all fragility fracture patients attended the FLS, as those with cognitive impairment and other severe comorbidities were excluded, this limits the generalizability of the service to all fragility fracture patients. An extension of this study design is to compare events post-fracture for patients who attend a hospital with an FLS vs without an FLS. Such studies have shown impressive reductions in refracture rates of 40–56% [48, 49]. In one study, only 103/515 (20%) attended the FLS clinic, suggesting any observed differences in fracture rates was unlikely to be driven by the FLS. Attendance was higher in the Dutch study 67.8% with no difference in refracture rate by attendee status, which is unexpected if the FLS is hypothesised as the cause for fracture reduction [49]. This study also showed a 43% reduction in mortality in women even though only 50% of those attending the FLS group were prescribed bisphosphonates, suggest AOM could only partly explain the mortality effect.

Systematic reviews have confirmed that patient/physician education strategies are ineffective [47, 50, 51] and identified critical issues in current literature such as using treatment initiation as the primary outcome [37] (a poor surrogate given the low adherence to therapy); non-contemporary or estimated control data [45, 52]; effect sizes based on randomised controlled trials for the drug [45, 46]; attendee vs non-attendee designs which are subject to significant selection bias, left censoring/immortal time bias [47, 53]. The magnitude of the effect of these biases is evidenced by the reported range of fracture reduction from 7 to 80%. A literature review that included all types of study, irrespective of methodological issues presented above, demonstrated significant increases in BMD testing, treatment initiation, adherence, refracture and mortality [54].

## Conclusion

The rationale for FLS efficacy is sound. There is no equipoise that giving an AOM to the high-risk patient will reduce fracture risk. There is ample supporting evidence for fracture efficacy from RCTs, including in the post-fracture care setting [55]. So, does the rationale for research activities to demonstrate the benefit of an FLS need to be reviewed? The urgent question for patients, clinicians and policymakers is whether the FLS care pathway can deliver the expected benefits from effective secondary prevention in their locality. FLSs often operate in complex local healthcare system relying on the significant interplay between people, equipment, processes and institutions as well as competing priorities, resources and reimbursement. Most complex services require active service improvement to adapt the service to become effective and sustainable based on local healthcare characteristics; one size does not fit all [56]. This requires an evidence base focussing on implementation and quality improvement rather than randomisation and comparison.

**Fig. 2:**
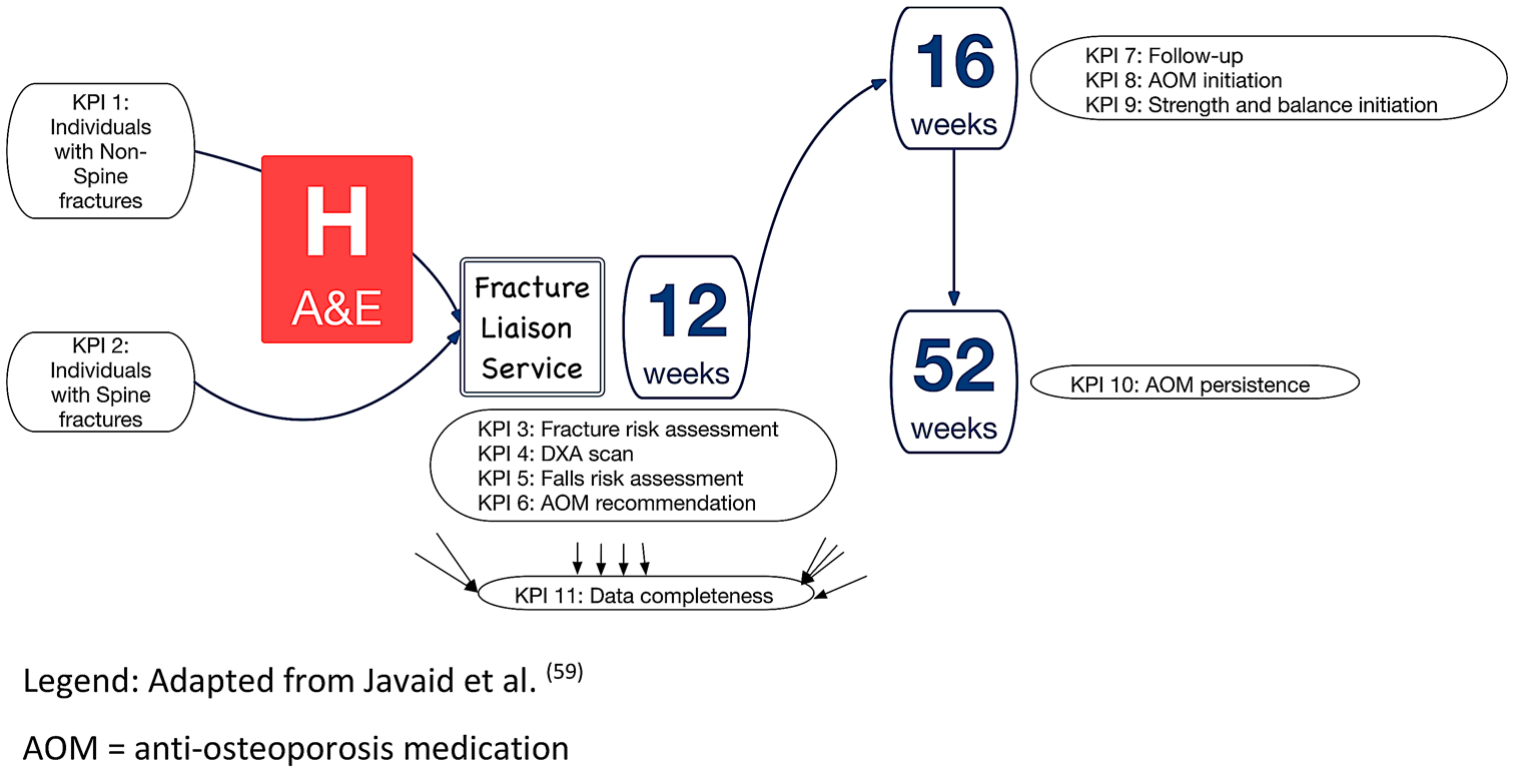
IOF/FFN/NOF indicator Set

Further, an FLS can only be effective if patients engage and adhere to each step of the pathway. Several patent level factors have been identified that need to be addressed by FLSs [57]. To achieve this, FLSs need to be adequately funded both in scale and duration to establish care pathways adapted and optimised to their local healthcare environment, a policy imperative. To achieve this, FLSs need to critically review their performance to identify areas for improvement, develop models of service changes and then analyse the impact of the service change on patient and process outcomes [56]. Quality Improvement capability, capacity and delivery should be included as another specification for FLSs [17]. The availability of data to inform service performance is fundamental to the process of Quality Improvement. Two indicator sets are available that benchmark services: on an organisational level, the IOF Capture the Fracture Best Practice Framework [58]; and on at the patient level, the IOF/FFN/NOF key performance indicator set [59] (Fig. 2). A limitation of these indicators is that they are primarily focussed on effectiveness and do not address efficiency or patient experience, areas for future research.

**Fig. 3:**
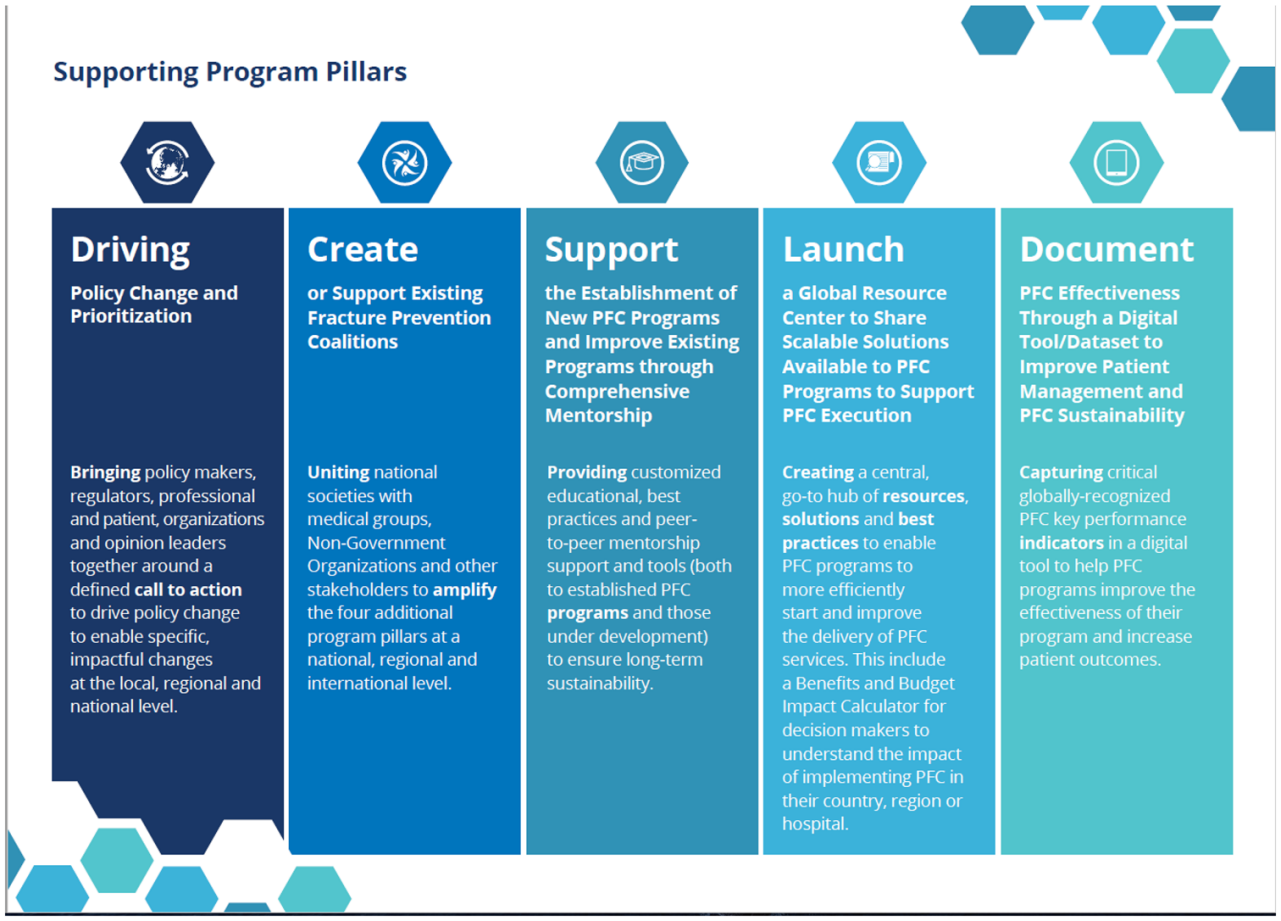
The capture the fracture partnership structure

Providing indicator sets only goes so far to improve performance. Since 2016, the UK FLSDB has been providing real-time performance data for participating FLSs [60, 61], which has resulted in modest improvements. International calls to action and patient charters have been used to increase political and patient awareness. In June 2020, the ambitious Capture the Fracture Partnership was launched to bring together global expertise in secondary prevention across pillars of policy, coalitions, mentorship, scalable solutions and digital tools (Fig. 3). The Capture the Fracture^®^ partnership, between the International Osteoporosis Foundation, the University of Oxford, Amgen and UCB, aims to support the broader implementation of FLS and other PFC related programs, to reduce the incidence of hip and vertebral fractures due to osteoporosis by 25% by the year 2025. Further academic work should focus on providing the evidence for optimising political prioritisation, local funding and sustainable delivery of effective and efficient FLSs and other PFC models with good patient experience. Hence, every patient after a fragility fracture receives secondary fracture prevention management to maintain physical and mental health, independence and dignity.
